# Enlarged perivascular space mimicking mesencephalothalamic cystic tumor

**Published:** 2014-10-06

**Authors:** Güliz Yılmaz, Süha Akpınar

**Affiliations:** Department of Radiology, School of Medicine, Near East University, Nicosia, North Cyprus, Turkey

**Keywords:** Perivascular Space, Magnetic Resonance Imaging, Cystic Mass

Perivascular spaces (PVSs) of the brain, also known as Virchow-Robin spaces are pial-lined interstitial fluid-filled structures that surround the small vessels as they extend into the brain parenchyma. PVSs, which are smaller than 2 mm can be seen in all age groups.^1^^,^^2^ Occasionally, PVSs may become large, cause mass effect and be misdiagnosed as a cystic neoplasm. These giant spaces are most common in the mesencephalothalamic region.^2^^,^^3^ The locations of PVSs are classified in three types. Type I occurs in the basal ganglia areas and anterior perforated substance. Type II appears in the white matter at the high convexities and type III is seen in the midbrain.^2^ PVSs are isointense to cerebrospinal fluid (CSF) at all sequences of magnetic resonance imaging (MRI) and have no contrast enhancement.^1^^,^^2^ The surrounding brain parenchyma of the PVSs usually has normal signal intensity.^1^ The top differential diagnoses of dilated PVSs are lacunar infarctions, cystic neoplasms, neuroepithelial cysts and infectious cysts.^1^^,^^2^

A 59-year-old woman was examined by the cranial MRI with the initial diagnosis of intracranial mass. There was a multilobular cystic mass in the mesencephalothalamic region that was isointense to CSF at all sequences and had no contrast enhancement or restricted diffusion. There was mild focal mass effect upon the third ventricle on the midline. There was also enlargement of the lateral and third ventricles (Figures 1 and 2). Recognition of giant PVSs location and characteristic MRI findings helps in differentiating them from neoplasms or other diseases.

**Figure 1:**
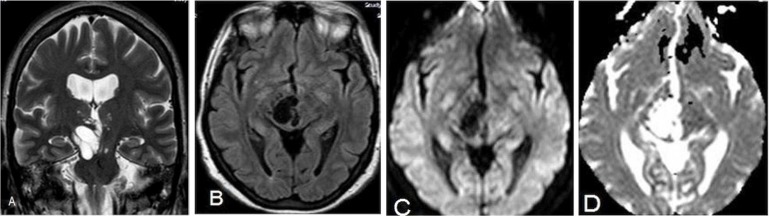
Coronal T2-weighted (W) (A) and axial fluid-attenuated inversion recovery (B) sequences magnetic resonance (MR) images demonstrate multilobular cystic mass isointense to cerebrospinal fluid in the mesencephalothalamic region. Coronal T2-W (A) MR image reveals the mild focal mass effect upon the third ventricle. Diffusion-weighted image (C) and apparent diffusion coefficient maps (D) show no restricted diffusion

**Figure 2:**
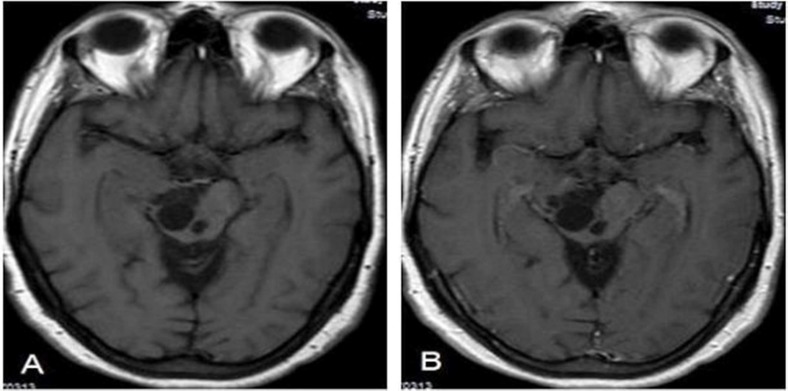
Axial T1-weighted (W) (A) and axial contrast-enhanced T1-W (B) magnetic resonance images reveal that there is no pathological contrast enhancement

## Notes

**How to cite this article:** Yılmaz G, Akpınar S. Enlarged perivascular space mimicking mesencephalothalamic cystic tumor. Iran J Neurol 2014; 13(4): 253-4.
